# Average Accumulative Based Time Variant Model for Early Diagnosis and Prognosis of Slowly Varying Faults

**DOI:** 10.3390/s18061804

**Published:** 2018-06-03

**Authors:** Funa Zhou, Ju H. Park, Chenglin Wen, Po Hu

**Affiliations:** 1 School of Computer and Information Engineering, Henan University, Kaifeng 475004, China; hupo4210@163.com; 2 Department of Electrical Engineering, Yeungnam University, 280 Daehak-Ro, Kyongsan 38541, Korea; 3 School of Automatic, Hangzhou Dianzi University, Hangzhou 310018, China; wencl@hdu.edu.cn

**Keywords:** early detection, fault prognosis, RUL prediction, average accumulative, error correction

## Abstract

Early detection of slowly varying small faults is an essential step for fault prognosis. In this paper, we first propose an average accumulative (AA) based time varying principal component analysis (PCA) model for early detection of slowly varying faults. The AA based method can increase the fault size as well as decrease the noise energy. Then, designated component analysis (DCA) is introduced for developing an AA-DCA method to diagnose the root cause of the fault, which is helpful for the operator to make maintenance decisions. Combining the advantage of the cumulative sum (CUSUM) based method and the AA based method, a CUSUM-AA based method is developed to detect faults at earlier times. Finally, the remaining useful life (RUL) prediction model with error correction is established by nonlinear fitting. Once online fault size defined by detection statistics is obtained by an early diagnosis algorithm, real-time RUL prediction can be directly estimated without extra recursive regression.

## 1. Introduction

Abnormal monitoring and efficient tolerant control of large scale systems is significant and crucial since it can guarantee safety and economic production [1,2,3,4,5,6,7,8,9,10,11,12]. For that reason, system abnormal monitoring has received much attention in theoretical and application research areas [1,2,3,4,5,6,7,8]. In general, three steps are required to secure the safety and economic production: abnormal detection, fault pattern recognition, and fault prognosis [1,2]. The first two steps are the necessary means to secure safe production by regular maintenance and repairing at certain time intervals of service time. It can alarm systems abnormal to the operator. Condition-based maintenance is one of the necessary means to secure economic production [2]. However, either abnormal detection or fault pattern recognition can not tell the operator whether a fault will occur and how long will the system be significantly affected, which is the foundation of condition-based maintenance.

**Remark** **1.** *In this paper, “Remaining Useful Life (RUL)” refers to the degrading time between fault- detected points and the point where faults occurred will significantly impact safety or the ability to complete a mission.In addition, the failure time is also defined in statistical means when the fault is statistically significant.*

In this section, the existing methods to detect slowly varying faults earlier is firstly overviewed, and then followed with the RUL prediction method overview. The data-driven fault diagnosis method is more applicable in the engineering field since only historical observation data are required, while the precise system model and expert knowledge of the system are not available [8,13,14,15,16,17,18,19,20,21,22,23,24,25]. Machine learning and statistical feature extraction based methods are the most common used ones of data driven fault diagnosis methods. Artificial neural network (ANN) and support vector machine (SVM) are the representative machine learning methods used in fault diagnosis [15,26,27]. However, a large number of historical faulty observations are required to establish a learning modeling of fault diagnosis, and it can not avoid the local optimal problem caused by unsuitable initial values. Principal component analysis (PCA) and partial least square (PLS) are the representative multivariate statistical methods used in data driven fault diagnosis [8,13,14,15,17,18,19,20,21,22]. These methods are more popular since only normal historical observation is required to establish PCA or PLS based abnormal detection models. However, the pattern compounding effect of PCA makes it difficult to diagnose the root cause of the fault. Designated component analysis (DCA) projects the observation to a designated pattern defined by the fault-symptom relation, so it can determine which failure component causes the abnormality [22,27,28]. These data-driven fault diagnosis methods have been widely used in the engineering field. If they are combined with data-driven RUL prediction technologies, fault prognosis approaches without physical models are developed [19,29,30]. Traditional fault diagnosis can only detect the failure moment of the system. For the purpose of fault prognosis, it is expected to detect fault trends as early as possible, and we call it early fault detection. Early detection of slowly varying small faults is an essential step to develop an efficient fault prognosis method [2,29,30].

**Remark** **2.** *In this paper, “failure moment” refers to the point when system is significantly affected by the fault occurring.*

Existing early detection methods of slowly varying small faults can be categorized into two classes: model based methods and data-driven methods [3,4]. Inaccurate parameters of a physical model can significantly affect the efficiency of model based early detection of small slowly varying faults. For the purpose of developing a data-driven small fault detection method, many necessary techniques have been used, such as a filter-based method, exponential weighted moving average (EWMA) based method and a cumulative sum (CUSUM) based method [23,24,25,26,31,32,33,34,35,36].

Filter based methods include moving average filter, median filter, wavelet transform (WT) filter, Hilbert–Huang transform (HHT), fast Fourier transform (FFT), short time Fourier transform (STFT), etc. [27]. These methods share the similarity that they all increase the signal-to-noise rate (SNR) of the fault signal by only decreasing the energy of the noise without increasing the the fault size. Although many diagnosing methods have been used as the preprocessing method in the case when small faults occurred, most fault diagnosis methods were invalid as long as SNR of the fault signal still does not increase to a larger value because of the insensitivity of these methods to rather small faults.

Traditional multivariate statistical analysis methods use only the current observation without considering the history information. Thus, the traditional control chart is less sensitive to small faults. To overcome this deficiency, both CUSUM and EWMA based methods take the cumulative sum of the fault size in order to increase SNR of the fault signal [26,30,35]. The main idea of CUSUM-PCA follows as: once the statistics value beyond the threshold defined in advance, make the cumulative sum of the fault size defined by the deviation between the statistics value and the threshold, which can be called fault size cumulating. After the CUSUM process, it can be seen that the fault size at the current time is updated by a cumulative fault size based on all fault sizes at previous times, and CUSUM based control chart can be used as an alternative choice of the traditional control chart [26,30]. Different from the CUSUM method, EWMA uses a weighting coefficient in the cumulative sum step [35]. Compared with CUSUM, EWMA can be more sensitive to small faults as long as the weight coefficient is properly selected. However, the weighting coefficient is usually selected by experience, which implies that it is not a universal value for different fault cases in different application areas.

For the RUL prediction stage following the early detection of small slowly varying faults, two kinds of RUL prediction can be generally used: a state space modeling based method and a statistical modeling based method.

For model based RUL prediction, if there is an exact damage precursor determined by experienced experts and the fault size itself is observable, then a damage precursor based RUL prediction method can be used [33,34,35,36,37,38]. However, the accuracy of the precursor greatly affects the prediction error. Furthermore, fault size can not be observed directly. In most cases, it is difficult to establish an appropriate damage precursor. In [38], a defect propagation model depicted in the form of a mechanistic model is used to predict the RUL of bearing [38]. The stiffness model or a crack growth law based physical relationship is adopted to predict the RUL of mechanical machine [19,39,40,41]. These methods share the same shortcomings: (1) physical model of fault propagation is required; (2) fault degradation process can be directly observed. In general, an accurate defect propagation model is difficult to obtain, which can significantly decrease the RUL prediction efficiency.

In the case when the fault propagation model for an exact damage precursor is unavailable, some state space model based prediction methods can be used, such as the Kalman filter based method, the particle filter based method, and the grey model based method [39,40,41,42]. Brown motion with a constant drift can be used as the state space model [43] for RUL prediction. In [44], Wang and Tsui use linear Brownian motion with adaptive drift as the physical model for RUL prediction with the assumption of inverse Gaussian distribution. In addition, the Kalman filter is adopted to update the drift of Brownian motion model.The innovation of [44] is that posterior drift previously was incorporated into the estimation of drift coefficient at the current time. For the case when the degradation of the faulty system is nonlinear, Si et al. established a [45,46] nonlinear Brownian motion with adaptive drift to get a more accurate RUL prediction method. However, the RUL prediction methods listed in literature [43,44,45,46] are all two-stage methods. These methods share the same deficiency with other model based methods that an accurate physical model is always unavailable. The reason is that the accuracy of drift coefficient for Brown motion model identification will significantly affect the accuracy of the final RUL prediction. On the other hand, an expert system (ES) based method is usually not a first choice for RUL prediction since ES inference and prediction may result in relatively large deviation when adverse knowledge exists in the actual engineering system [47]. A data-driven approach for RUL prediction has been investigated [38]. It is noted that data-driven techniques such as ANN or PCA can be used to establish a data-driven RUL prediction model when the physical model of system degradation process is inaccurate [38]. Thus, the statistical modeling based method is used in this paper for the case when there is no accurate physical model.

Statistical modeling based methods establish the RUL estimation models by fitting the model to historical data under a statistical model without relying on any physical principle [48]. The basic idea of statistical modeling based method is to use historical observed data and statistical models to estimate the RUL [48,49,50]. In general, statistical modeling based methods can be categorized into two classes: models relying on directly observed state information and those that do not. As is already known, there is usually very little failure data. Thus, it is a good alternative to predict RUL based on directly observed state process. The set-up of the failure threshold is often a decision based on the analysis of historical data or the recommended standards provided by expert. In this paper, the observation is assumed to be Gaussian distribution only to determine the threshold through a hypothesis test of some multivariate statistical analysis method. The common used RUL prediction model includes regression-based models, Gamma processes based models, machine learning based models, etc. Regression-based methods establish a regression model for performance parameters first, and then future performance parameters can be predicted by the established regression model autoregressive (AR) or vector autoregressive (VAR) based method, and the time series analysis based method [42,51,52,53,54]. Furthermore, a wide variety of advanced computational techniques such as SVM, ANN, and dynamic Bayesian networks have been used for estimating RUL of industrial systems [42]. ANN based RUL prediction models require a large number of fault degradation signals, which is usually impractical, even when the acceleration life test is used. Moreover, the problem of local optimal can not be avoided. Even fewer fault data are required for SVM to learn a prediction model, and more than one set of faults is necessary.

In the case when only one set of fault data is available, a statistical prediction model such as AR or VAR can be used to predict the fault size in the future [19,30,37]. RUL prediction results based on AR or VAR can only predict impending failure in limited steps ahead [19,30], which does not coincide with the intention of predicting the failure time as early as possible to provide sufficient time for maintenance decision-making. To overcome these limitations, prediction based on multi-step regressive regression is required to estimate the RUL [19]. Due to uncertainty propagation, accumulated prediction error may be a large value during the recursive regression process. In addition, two steps of fault prediction followed by RUL estimation does not coincide with real-time requirements of online prognosis. Finally, a prediction model based on PCA feature extraction can only predict RUL of the system rather than RUL of a specific component of the system.

The main innovations of this paper are as follows: since early diagnosis of slowly varying faults is the fundamental basis of RUL prediction, a more effective AA based time variant model for early fault detection is proposed in the first part of the paper, which can accumulate the fault size as well as decrease the noise of the observation. By using this method, variance of the statistics is much smaller, which is more advantageous for combining a AA based method with a CUSUM based method for detecting fault trends earlier. Then, for the purpose of telling the maintenance department which component should be maintained, RUL prediction for specific components based on the cumulative sum-accumulated average-designated component analysis (CUSUM-AA-DCA) method is further developed. Finally, to illustrate the final goal of early fault diagnosis, the RUL prediction model of exponential regression with error correction is established by nonlinear fitting in the case when early fault trends are diagnosed. The statistics value to describe the performance index and RUL are the independent variable and the dependent variable of the RUL prediction model, respectively. Once the online detection statistics is obtained by an early detection algorithm, the real-time RUL can be predicted by this model without extra multi-step recursive regression.

This paper is organized as follows: Section 2 reviews PCA and DCA based fault detection methods. In Section 3, AA based early diagnosis methods for slowly varying small faults are proposed. In Section 4, an RUL prediction model with error correction is proposed by nonlinear fitting. Section 5 illustrates the numerical simulation of our proposed algorithm to show its efficiency. Section 6 provides the conclusions of this work.

## 2. Review of PCA and DCA

### 2.1. PCA

In the case when *m* sensors are equipped in the system and *n* observation samples of each sensor are collected, $Y_{0}\in R^{n\times m}$ is used to represent the historical normal observation. The following equation illustrates the PCA decomposition of $Y_{0}$

(1) $$\begin{array}{c} T_{0j}=Y_{0}^{T}P_{j}, \end{array}$$

(2) $$\begin{array}{c} Y_{0}=PT_{0}+E_{0}, \end{array}$$

where $P_{j}\in R^{m\times 1}$ is the loading vector defined by eigenvector of $\Sigma_{0}=\frac{1}{n}Y_{0}^{T}Y_{0}$, $T_{0j}$ is the *i*th score vector, $P=\left[P_{1}\;P_{2}\;\cdots P_{\upsilon }\right]\in R^{m\times \upsilon }$ is the first υ columns of the loading matrix, and υ is the number of key principal components, and $E_{0}=\sum_{j=\upsilon +1}^{m}P_{j}T_{0j}$ is the corresponding residual matrix.

Once the square prediction error (SPE) statistics defined in Equation (2) go beyond the control limit, the system is inferred to be abnormal in the sense of the hypothesis test:

(3) $$SPE\equiv {∥E∥}^{2}=Y\left(I-PP^{T}\right)Y^{T}.$$

### 2.2. DCA

The pattern compounding effect of PCA actually makes it difficult to distinguish fault patterns corresponding to a specific component, thus it may fail to reach any practical diagnostic conclusion that the engineer operator required [27,28]. Although a fault pattern recognizing method can be used after detection, diagnosis results by this two step method may be not in real time. Thus, timely fault maintenance strategy can not be made, which may lead to disastrous accidents.

As is already known, PCA projects observation to the loading vector defined by the historical normal data. Different from PCA, DCA projects observation to the designated patterns $D_{j}$ defined as below:

(4) $$\begin{array}{c} D_{j}={\left[d_{j1},d_{j2},\cdots ,d_{jm}\right]}^{T}, \end{array}$$

where

(5) $$d_{js}=\left\{\begin{array}{ll} 1, & j\mathrm{th}\;\mathrm{variation}\;\mathrm{pattern}\;\mathrm{shows}\;s\mathrm{th}\;\mathrm{symptom},\\ 0, & j\mathrm{th}\;\mathrm{variation}\;\mathrm{pattern}\;{\mathrm{doesn}}^{’}t\;\mathrm{shows}\;s\mathrm{th}\;\mathrm{symptom}. \end{array}\right.$$

Compute the designated components (DC) via the following equation

(6) $$\begin{array}{c} W_{0j}=Y_{0}^{T}D_{j}, \end{array}$$

where $W_{0j}$ is the designated component vector corresponding to $D_{j}$.

Similar to PCA, DCA decomposition of $Y_{0}$ can be described in Equation (7) [22,27]:

(7) $$\begin{array}{c} Y_{0}=\sum_{j=1}^{l}D_{j}W_{0j}+E_{0}, \end{array}$$

where l≤m is the number of designated patterns.

A hypothesis test of normal distribution can be used as a tool to determine the control limit of each designated component.

**Remark** **3.** *The designated pattern can be defined by experts’ experience or by machine learning to a specific faulty observation.*

## 3. AA Based Early Diagnosis of Slowly Varying Small Faults

An early detection method is necessary not only to detect the fault occurring moment, but also to predict the RUL of the system before it is broken down. In this part, an AA technique is proposed to increase the fault size as well as to decrease the noise energy, thus SNR of the fault signal can be significantly enhanced, which is beneficial for early detection of slowly varying faults.

### 3.1. AA Based Time Variant PCA for Early Abnormal Detection

As stated in Section 1, the CUSUM based method uses the cumulative sum of fault size to enhance the SNR of the fault signal. In the case when fault size can not be directly observed, statistics of PCA such as SPE can be defined as an indirect fault size to be cumulated in CUSUM-PCA [25]. However, it does not take noise energy decreasing into consideration, so the efficiency of early abnormal detection is not satisfactory.

In this subsection, with our intention to decrease the noise variance of the observation as well as to cumulate the fault size at each sample time, a new AA based time variant PCA model is proposed for early detection of fault evolved slowly.

**Remark** **4.** *It can be proved that, after the AA process, the noise variance of the observation can be decreased as well as the fault size can be increased. On the other hand, the average accumulative observation at each time is not identical independent distribution (i.i.d.) samples any more. To overcome this limitation, time variant AA model can be established as long as there is adequate number of historical normal data.*

The detailed algorithm includes seven steps:

**A. Offline modeling**

**Step 1:** Assume there are nN samples of normal historical observation. Denote ${\bar{Y}}_{0}\in R^{m\times n\times N}$ as the stacked normal historical observation, where *m* is the number of observation variables involved in the system, *n* is the number of samples of each variable, and *N* is equal to the number of samples of online observation.

**Step 2:** Compute *N* set of average accumulation ${\hat{Y}}_{0}^{\left(k\right)}\in R^{m\times n}$ from

(8) $$\begin{array}{c} {\hat{Y}}_{0}^{\left(k\right)}\left(i,j\right)=\frac{1}{k}\sum_{s=1}^{k}{\bar{Y}}_{0}\left(i,j,s\right),\;\;k=1,2,\cdots ,N, \end{array}$$

where ${\bar{Y}}_{0}\left(i,j,s\right)$ is the element of ${\bar{Y}}_{0}$, ${\hat{Y}}_{0}^{\left(k\right)}\left(i,j\right)$ is the element of ${\hat{Y}}_{0}^{\left(k\right)}$, and the superscript (k) denotes the *k*th set of average accumulative observation.

**Step 3:** Establish *N* anomaly detection models based on each ${\hat{Y}}_{0}^{\left(k\right)}\left(k=1,2,\cdots ,N\right)$ from

(9) $$\begin{array}{c} {\hat{Y}}_{0}^{\left(k\right)}=T_{0}^{\left(k\right)}{P^{\left(k\right)}}^{T}+E^{\left(k\right)},\;\;\left(k=1,2,\cdots ,N\right), \end{array}$$

where $P^{\left(k\right)}$ is the first υ(k) columns of the loading matrix, υ(k) is the number of key principal components for the *k*th PCA model, $T_{0}^{\left(k\right)}$ is the score matrix, and $E^{\left(k\right)}$ is the residual matrix.

**Step 4:** The statistics of SPE are used for anomaly detection. The control limit $U\hat{C}L\left(k\right)$ can be determined by

(10) $$\begin{array}{c} U\hat{C}L\left(k\right)=\theta_{1}\left(k\right){\left[\frac{h_{0}\left(k\right)C_{\alpha }\sqrt{2\theta_{2}\left(k\right)}}{\theta_{1}\left(k\right)}+\frac{\theta_{2}\left(k\right)h_{0}\left(k\right)\left(h_{0}\left(k\right)-1\right)}{{\left(\theta_{1}\left(k\right)\right)}^{2}}+1\right]}^{1/h_{0}\left(k\right),} \end{array}$$

where

(11) $$\begin{array}{ccc} \theta_{l}\left(k\right) & = & \sum_{j=\upsilon (k)+1}^{m}{\left(\lambda_{j}\left(k\right)\right)}^{l},\;\;l=1,2,3, \end{array}$$

(12) $$\begin{array}{ccc} h_{0}\left(k\right) & = & 1-\frac{2\theta_{1}\left(k\right)\theta_{3}\left(k\right)}{3{\left(\theta_{2}\left(k\right)\right)}^{2}}. \end{array}$$

$C_{\alpha }$ is the α-quantile of normal distribution N(0,1), and $\lambda_{j}\left(k\right)$ is the eigenvalue of $\Sigma_{0}^{\left(k\right)}=\frac{1}{n}Y_{0}^{(k)T}Y_{0}^{\left(k\right)}$.

**B. Online detection**

For online detection, it is assumed that the online data $Y\in R^{n\times m}$ is defined as

(13) $$\begin{array}{c} Y\left(i,j\right)=Y_{0}\left(i,j\right)+F\left(i,j\right), \end{array}$$

where $Y_{0}\left(i,j\right)$ is the online normal observation of the *j*th variable at sample time *i*, and F(i,j) is the observation variation of the *j*th variable at sample time *i* when the system is abnormal.

Online early fault detection of slowly varying small faults follows as Steps 5–7:

**Step 5:** Compute the average accumulative sum of the previous *k* samples from

(14) $$\hat{Y}\left(k,j\right)=\frac{1}{k}\sum_{i=1}^{k}Y_{0}\left(i,j\right)+\hat{F}\left(k,j\right),$$

where

(15) $$\hat{F}\left(k,j\right)=\frac{1}{k}\sum_{i=1}^{k}F(i,j).$$

The signal-to-noise-rate (SNR) of $\hat{F}\left(k,j\right)$ is defined as

(16) $$\begin{array}{c} S\hat{N}R\left(k,j\right)=\frac{\hat{F}\left(k,j\right)}{\hat{\sigma }\left(k,j\right)}, \end{array}$$

where $\hat{\sigma }\left(k,j\right)$ is the noise variance of $\hat{F}\left(k,j\right)$.

**Remark** **5.** *It should be noted that the fault signal is assumed to be a deterministic signal generated by performance degradation of a component in the system.*

After average accumulative summing, it can be proved that

(17) $$S\hat{N}R\left(k,j\right)\geq SNR\left(k,j\right),$$

where SNR(k,j) is the SNR of F(k,j), which is defined as

(18) $$\begin{array}{c} SNR\left(k,j\right)=\frac{{\left|F(k,j)\right|}^{2}}{\sigma^{2}}. \end{array}$$

The detailed proof is as follows:

(19) $$\begin{array}{c} S\hat{N}R\left(k,j\right)=\frac{{∥\hat{F}(k,j∥}^{2}}{\hat{\sigma }\left(k,j\right)}=\frac{\frac{1}{k}\sum_{i=1}^{k}{\left|F(i,j)\right|}^{2}}{\mathrm{var}(\frac{1}{k}\sum_{i=1}^{k}Y(i,j))}=\frac{\frac{1}{k}\sum_{i=1}^{k}{\left|F(i,j)\right|}^{2}}{\mathrm{var}(\frac{1}{k}\sum_{i=1}^{k}Y_{0}(i,j))}=\frac{\frac{1}{k}\sum_{i=1}^{k}{\left|F(i,j)\right|}^{2}}{\frac{\sigma^{2}}{k}},\\ \;\;\;\;=\left\{\begin{array}{ll} 0, & k\in S_{n},\\ k\frac{\frac{1}{k}\sum_{i=1}^{k}{\left|F(i,j)\right|}^{2}}{\sigma^{2}}, & k\in S_{a}, \end{array}\right. \end{array}$$

where $S_{n}=\underset{system\;is\;normal\;at\;k}{\arg}\left\{k\right\}$ and $S_{a}=\underset{system\;is\;abnormal\;at\;k}{\arg}\left\{k\right\}$, var(x) denotes the variance operation of *x*.

Since the noise variance decreases 1/k times, we can prove that $S\hat{N}R\left(k,j\right)\geq SNR\left(k,j\right)$ as long as $\sum_{i=1}^{k}{\left|F(i,j)\right|}^{2}\geq {\left|F(k,j)\right|}^{2}$.

**Figure 1:**
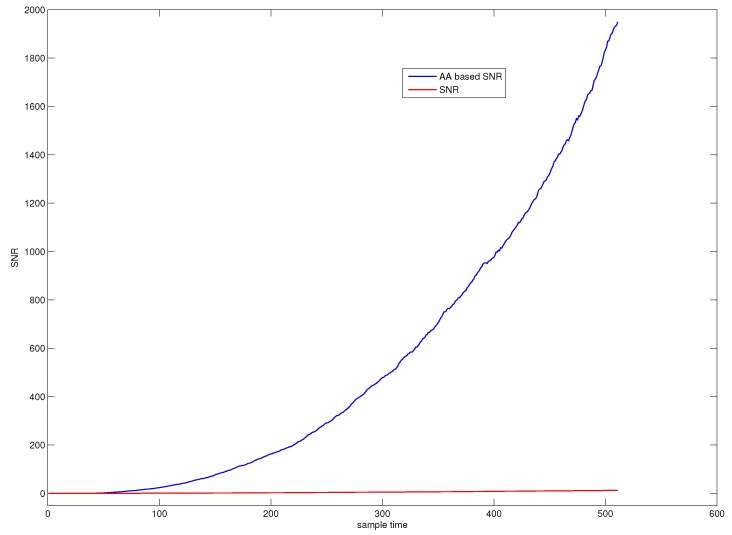
SNR of a fault observation.

Figure 1 illustrates the SNR of a fault observation, where the red line represents the original SNR and the blue line represents the SNR computed by average accumulation. The figure indicates that average accumulative technique can greatly enhance the SNR of the small slowly varying fault buried in noise.

**Remark** **6.** *The key idea of small fault detection is to increase the fault size and to decrease the noise energy. In such a way, the control limit can be decreased and the fault size can be increased.*

**Step 6:** Project $\hat{Y}\left(k\right)=\left[\hat{Y}\left(k,1\right),\hat{Y}\left(k,1\right),\cdots \hat{Y}\left(k,m\right)\right]$ to the principal model defined in Equations (9) and (10). Compute the online SPE value from

(20) $$S\hat{P}E\left(k\right)={∥\hat{E}\left(k\right)∥}^{2}=\hat{Y}\left(k\right)\left(I-P^{k}{P^{k}}^{T}\right){\hat{Y}}^{T}\left(k\right),$$

where $\hat{E}\left(k\right)$ is the residual vector when $\hat{Y}\left(k\right)$ is projected to the principal component space spanned by $P^{\left(k\right)}$.

**Step 7:** If $S\hat{P}E\left(k\right)$ violates the control limit of the *k*th principal model, the system is abnormal at time *k*; otherwise, the system is normal.

### 3.2. AA Based Time Variant DCA for Early Fault Diagnosis

AA-PCA can be used for early detection of slowly varying faults, but it is only efficient in early detection of system abnormality. The pattern compounding effect of PCA makes it difficult to recognize which component failure is the root cause [27,28]. In general, root causes of different faults correspond to different components of the system. Especially, in the case when multiple faults are involved in the system, each fault may occur from different times. On the other hand, fault prognosis is in essence the RUL estimation of every component, which can tell the engineering operator how long a specific component will be maintained or replaced. Thus, early separate diagnosis of every slowly varying fault component is required for fault prognosis. In this subsection, an average accumulative based time variant DCA model for early diagnosis is proposed.

The algorithm includes the following five steps:

**A. Offline modeling**

**Step 1:** Obtain *N* sets of average accumulation of historical observation ${\hat{Y}}_{0}^{\left(k\right)}$ from Equation (8).

**Step 2:** Project the average accumulated historical data ${\hat{Y}}_{0}^{\left(k\right)}$ to the designated pattern $D_{j}\;\left(j=1,2,\cdots ,l\right)$ defined in advance according to the fault-symptom knowledge determined by experts or data driven machine learning algorithms:

(21) $${\hat{W}}_{0j}^{\left(k\right)}=D_{j}^{T}{\hat{Y}}_{0}^{{\left(k\right)}^{T}}\;\left(k=1,2,\cdots ,N,\;j=1,2,\cdots ,l\right).$$

**Step 3:** Establish *N* DCA fault diagnosis models.

Since ${\hat{W}}_{0j}^{\left(k\right)}$ is still normally distributed with zero mean, a designated component of normal observation should be located in the confidence interval of $\left(-3{\hat{\sigma }}_{j}\left(k\right),3{\hat{\sigma }}_{j}\left(k\right)\right)$ with the confidence level 99.7%, where ${\hat{\sigma }}_{j}\left(k\right)$ is the variance of ${\hat{W}}_{0j}^{\left(k\right)}$. The control limit for fault diagnosis can be computed by hypothesis testing via the following equations:

(22) $$U\hat{C}L_{j}\left(k\right)=3{\hat{\sigma }}_{j}\left(k\right),$$

(23) $$L\hat{C}L_{j}\left(k\right)=-3{\hat{\sigma }}_{j}\left(k\right).$$

**B. Online early diagnosis for slowly varying small faults**

**Step 4:** Project the average accumulated online data $\hat{Y}\left(k\right)$ to the designated pattern $D_{j}\;\left(j=1,2,\cdots ,l\right)$, where *k* is the current sampling time:

(24) $${\hat{W}}_{j}\left(k\right)=D_{j}^{T}{\hat{Y}}^{T}\left(k\right)\;\left(j=1,2,\cdots ,l,\;k=1,2,\cdots ,N\right).$$

**Step 5:** Comparing ${\hat{W}}_{j}\left(k\right)$ with the control limit of the *k*th DCA fault diagnosis model to evaluate whether the fault corresponding to $D_{j}$ has occurred in the system.

Instead of AA-PCA based time variant early fault detection, which can only detect whether the system is abnormal, the AA-DCA based time variant model for early diagnosis can effectively detect which component of the system is failure and the start point of each small fault can also be detected.

### 3.3. CUSUM-AA Based Early Diagnosis of Slowly Varying Small Faults

Even though the AA based method is efficient for slowly varying fault detection, more early small faults can not be well detected. On the other hand, the variance of the AA based statistics characterizing fault size is much smaller, which is beneficial for combining the AA based method with the CUSUM based method. Combining AA with CUSUM, a novel CUSUM-AA based method is developed in this subsection. It can be seen from Section 3.1 and Section 3.2 that cumulative sum-accumulated average-principle component analysis (CUSUM-AA-PCA) is similar to CUSUM-AA-DCA with the exception that the statistics used are SPE for CUSUM-AA-PCA and DC for CUSUM-AA-DCA, respectively. In order to save space in this paper, only the CUSUM-AA-DCA method for early diagnosis of slowly varying small faults is illustrated in detail. This method aims to diagnose slowly varying small faults earlier than AA-DCA.

There are six steps included in the CUSUM-AA-DCA algorithm:

**A. Offline modeling**

**Step 1:** Compute the average accumulation of historical observation ${\hat{Y}}_{0}^{\left(k\right)}$ via Equation (8).

**Step 2:** Compute the AA-based designated component ${\hat{W}}_{0j}^{\left(k\right)}$ from Equation (21).

**Step 3:** For early fault diagnosis of multiple slowly varying faults occurring at different times, CUSUM-AA based Shewhart control charts incorporating historical information for each DC is used as an alternative choice of traditional Shewhart control charts:

(25) $$\begin{array}{ccc} C{\hat{W}}_{0j}^{(k)+}\left(p\right) & = & \max[0,C{\hat{W}}_{0j}^{(k)+}\left(p-1\right)+{\hat{W}}_{0j}^{\left(k\right)}\left(p\right)-{\hat{\mu }}_{0j,w}\left(p\right)-\delta ], \end{array}$$

(26) $$\begin{array}{ccc} C{\hat{W}}_{0j}^{(k)-}\left(p\right) & = & \max[0,C{\hat{W}}_{0j}^{(k)-}\left(p-1\right)+{\hat{\mu }}_{0j,w}\left(p\right)-{\hat{W}}_{0j}^{\left(k\right)}\left(p\right)-\delta ], \end{array}$$

(27) $$\begin{array}{ccc} C{\hat{W}}_{0j}^{\left(k\right)}\left(p\right) & = & \max[C{\hat{W}}_{0j}^{(k)+}\left(p\right),C{\hat{W}}_{0j}^{(k)-}\left(p\right)], \end{array}$$

where $C{\hat{W}}_{0j}^{(k)+}\left(p\right)$ and $C{\hat{W}}_{0j}^{-}\left(p\right)$ are the upper and lower CUSUM designated component statistics, ${\hat{\mu }}_{0j,w}\left(p\right)$ is the mean of ${\hat{W}}_{0j}^{\left(k\right)}$, δ is usually a more tight threshold than that of the traditional method, such as one half of the traditional control limit [30]. It can be seen that the fault size beyond δ is added in a cumulative manner.

**Step 4:** Determine threshold $CU\hat{C}L_{j}$ as a new control limit of the *j*th designated component based on CUSUM-AA. The criterion with smallest miss detection rate is used to determine the new control limit. In fact, as long as δ is properly chosen to ensure the number of samples beyond δ is small enough, the CUSUM-AA based $CU\hat{C}L_{j}$ can be a rather small value. The ideal value of $CU\hat{C}L_{j}$ is zero.

**B. Online early diagnosis for slowly varying small faults**

**Step 5:** Project $\hat{Y}\left(k\right)$ to the designated pattern $D_{j}$ to obtain the average accumulative designated component ${\hat{W}}_{j}\left(k\right)$.

**Step 6:** Using the same means of cumulative sum with offline modeling to cumulate the online ${\hat{W}}_{j}\left(k\right)$ via the following Eqs.:

(28) $$\begin{array}{ccc} C{\hat{W}}_{j}^{+}\left(k\right) & = & \max[0,C{\hat{W}}_{j}^{+}\left(k-1\right)+{\hat{W}}_{j}\left(k\right)-{\hat{\mu }}_{0j,w}\left(k\right)-\delta ], \end{array}$$

(29) $$\begin{array}{ccc} C{\hat{W}}_{j}^{-}\left(k\right) & = & \max[0,C{\hat{W}}_{j}^{-}\left(k-1\right)+{\hat{\mu }}_{0j,w}\left(k\right)-{\hat{W}}_{j}\left(k\right)-\delta ], \end{array}$$

(30) $$\begin{array}{ccc} C{\hat{W}}_{j}\left(k\right) & = & \max[C{\hat{W}}_{j}^{+}\left(k\right),C{\hat{W}}_{j}^{-}\left(k\right)]. \end{array}$$

**Step 7:** Comparing $C{\hat{W}}_{j}\left(k\right)$ with the control limit $CU\hat{C}L_{j}$ to evaluate whether the fault corresponding to $D_{j}$ has occurred in the system.

The CUSUM-AA-PCA based early detection method for slowly varying faults can be developed in a similar manner.

## 4. RUL Prediction

Once the health state of the system is detected online, RUL of the current state should be estimated for decision-making of system maintenance. Here, the RUL prediction model based on historical fault data is established first. Then, online RUL prediction using the RUL prediction model can be obtained.

In this paper, we only consider those slowly varying faults with certain degradation rates.

### 4.1. Damage Precursor and RUL Prediction Model Based on Historical Fault Data

#### 4.1.1. CUSUM-AA-PCA Based System RUL Prediction Model

For sake of establishing an RUL prediction model, a degradation precursor based on early detection results of CUSUM-AA-PCA to historical faulty observation should be determined in the first step.

Firstly, early detection point $t_{start}$ of fault trends can be determined by the following criterion:

(31) $$t_{start}=\underset{k}{\arg\min}\left\{\begin{array}{c} CS\hat{P}E_{0}\left(k\right)\geq CU\hat{C}L,\\ CS\hat{P}E_{0}\left(s\right)\geq CU\hat{C}L\;k<s\leq k+5, \end{array}\right.$$

where $CU\hat{C}L$ is the control limit of CUSUM-AA-PCA model, and the subscript 0 represents the corresponding $CS\hat{P}E$ value of the historical fault observation.

Secondly, the failure point can be determined by the following criterion:

(32) $$t_{failure}=\underset{k}{\arg\min}\left\{\begin{array}{c} SPE_{0}\left(k\right)\geq UCL,\\ SPE_{0}\left(s\right)\geq UCL\;k<s\leq k+5, \end{array}\right.$$

where UCL is the control limit of PCA model established directly from the historical normal observation.

Thirdly, the evolution process between $t_{start}$ and $t_{failure}$ of the statistics for CUSUM-AA-PCA can be defined as the damage precursor from

(33) $$C\left(t\right)=CS\hat{P}E_{0}\left(t\right),\;\;\left(t=k-t_{start}+1,k-t_{start}+2,\cdots ,t_{failure}\right).$$

Define the RUL at *t* as

(34) $$\begin{array}{c} R\left(t\right)=t_{failure}-t. \end{array}$$

Then, using the damage precursor C(t) and the RUL at each $t\in [t_{\mathrm{start}},\;t_{\mathrm{failure}}]$, a nonlinear fit curve is developed to establish the rough RUL prediction model as below:

(35) $$\hat{R}=f\left(CS\hat{P}E_{0}\right).$$

Once SPE is used as a statistics to characterize the damage precursor, exponent function defined in the following equation is adopted to fit the curve of damage precursor:

(36) $$f\left(z\right)=a\times e^{bz}.$$

The prediction model proposed in this subsection can be used for online RUL prediction of the same fault as modeling.

**Remark** **7.** *As is already known, RUL based on accumulated damage usually has the form of exponent function. Since accumulated SPE is used to define fault size, there is no function choice problem because accumulated damage is used.*

Model error will inevitably result in online RUL prediction error. Thus, an error correction table can be developed in the modeling stage via the following criterion:

(37) $$E_{R}\left(CS\hat{P}E_{0}\left(k\right)\right)=R_{0}\left(CS\hat{P}E_{0}\left(k\right)\right)-f\left(CS\hat{P}E\left(k\right)\right).$$

The corrected prediction model can be illustrated as

(38) $$RUL\left(k\right)=f\left(CS\hat{P}E\left(k\right)\right)+E_{R}\left(CS\hat{P}E\left(k\right)\right),$$

where $CS\hat{P}E\left(k\right)$ is the online SPE value of CUSUM-AA-PCA, and it can be computed in a similar form to Equation (30) by replacing ${\hat{W}}_{i}\left(k\right)$ with $S\hat{P}E\left(k\right)$.

The common used AR or VAR prediction model characterizes the successive linear relation in local range [24]. It can only well predict the fault size in limited steps ahead, called PH steps. When the failure will come after PH steps, it can not directly predict the RUL. Multi-step regression in recursive form is required to predict how long the system will be failure. Therefore, the RUL prediction by VAR is not real-time.

Compared with the regression prediction model, the main advantage of our RUL prediction model is as follows:

(1) It can directly predict the RUL without extra recursive regression, thus it can insure ’real-time’ prediction of the RUL.
(2) The model established has the error correction term, so it can come to an accurate prediction.

#### 4.1.2. Online RUL Prediction

Once online $CS\hat{P}E\left(k\right)$ is obtained, the RUL at time *k* can be predicted online as

(39) $$RUL\left(k\right)=f\left(CS\hat{P}E\left(k\right)\right)+RE\left(CS\hat{P}E\left(k\right)\right)=a\times e^{b\times CS\hat{P}E\left(k\right)}+E_{R}\left(CS\hat{P}E\left(k\right)\right),$$

where $E_{R}\left(CS\hat{P}E\left(k\right)\right)$ is the error correction term when the online statistics value is $CS\hat{P}E\left(k\right)$.

The online error correction term $E_{R}\left(CS\hat{P}E\left(k\right)\right)$ can be determined by the following criteria:

(1) If there is a $CS\hat{P}E_{0}\left(k\right)$ such that $CS\hat{P}E_{0}\left(k\right)=CS\hat{P}E_{0}\left(k\right)$, then
  (40) $$E_{R}\left(CS\hat{P}E\left(k\right)\right)=E_{R}\left(CS\hat{P}E_{0}\left(k\right)\right).$$
(2) If there is no $CS\hat{P}E_{0}\left(k\right)$ such that $CS\hat{P}E_{0}\left(k\right)=CS\hat{P}E_{0}\left(k\right)$, then
  (41) $$E_{R}\left(CS\hat{P}E\left(k\right)\right)=E_{R}\underset{\;CS\hat{P}E_{0}\left(k\right)}{(\arg\min}\left(CS\hat{P}E_{0}-CS\hat{P}E\left(k\right)\right)).$$

### 4.2. DCA Based RUL Prediction of Each DC

Different from the ability of PCA to detect system abnormal without finding the root cause of the fault, DCA can diagnose multiple faults well corresponding to different components of the system. On the other hand, the system maintenance engineer wants to know exactly which component should be repaired, so it is more valuable to develop an RUL prediction model based on each DC of CUSUM-AA-DCA.

Replace $CS\hat{P}E_{0}\left(k\right)$ with $C{\hat{W}}_{0,i}\left(k\right)$ to get the starting point and failure point of the fault corresponding to $D_{j}$

(42) $$\begin{array}{c} t_{0j,start}=\underset{k}{\arg\min}\left\{\begin{array}{c} \left|C{\hat{W}}_{0,j}\left(k\right)\right|\geq CU\hat{C}L_{j},\\ \left|C{\hat{W}}_{0,j}\left(s\right)\right|\geq CU\hat{C}L_{j}\;k<s\leq k+5, \end{array}\right. \end{array}$$

where $CU\hat{C}L_{j}$ is the control limit of the the DC corresponding to $D_{j}$.

The failure point $t_{0j,failure}$ can be determined by the following criterion:

(43) $$\begin{array}{c} t_{0j,failure}=\underset{k}{\arg\min}\left\{\begin{array}{c} \left|W_{0,j}\left(k\right)\right|\geq UCL_{j},\\ \left|W_{0j}\left(s\right)\right|\geq UCL_{j}\;k<s\leq k+5, \end{array}\right. \end{array}$$

where $UCL_{j}$ is the upper control limit of DCA model established directly from the historical normal observation.

The damage precursor corresponding to $D_{j}$ can be computed as follows:

(44) $$\begin{array}{c} C_{j}\left(t\right)=C{\hat{W}}_{j0}\left(t\right),\;\left(t=k-t_{j0,start}+1,k-t_{j0,start}+1,\cdots ,t_{j0,failure}\right). \end{array}$$

RUL of the component corresponding to $D_{j}$ at *t* is defined as

(45) $$\begin{array}{c} R_{j}\left(t\right)=t_{j0,failure}-t_{j0,start}. \end{array}$$

Then, fitting curve of $C_{i}\left(t\right)$ and the RUL at each $t\in [t_{j0,\mathrm{start}},\;t_{j0,\mathrm{failure}}]$ can be developed as

(46) $$\begin{array}{c} {\hat{R}}_{j0}=a_{j}+b_{j}\times e^{c_{j}C{\hat{W}}_{j,0}}. \end{array}$$

The corresponding error correction table can also be determined via the following relationship

(47) $$\begin{array}{c} E_{j,R}\left(C{\hat{W}}_{j,0}\left(k\right)\right)=R_{j0}\left(C{\hat{W}}_{j,0}\left(k\right)\right)-f\left(C{\hat{W}}_{j,0}\left(k\right)\right). \end{array}$$

Once online $C{\hat{W}}_{j}\left(k\right)$ is obtained, the RUL at time *k* can be predicted online as

(48) $$\begin{array}{c} RUL_{j}\left(k\right)=a_{j}+b_{j}\times e^{c_{j}C{\hat{W}}_{j}\left(k\right)}+E_{j,R}\left(C{\hat{W}}_{j}\left(k\right)\right). \end{array}$$

**Figure 2:**
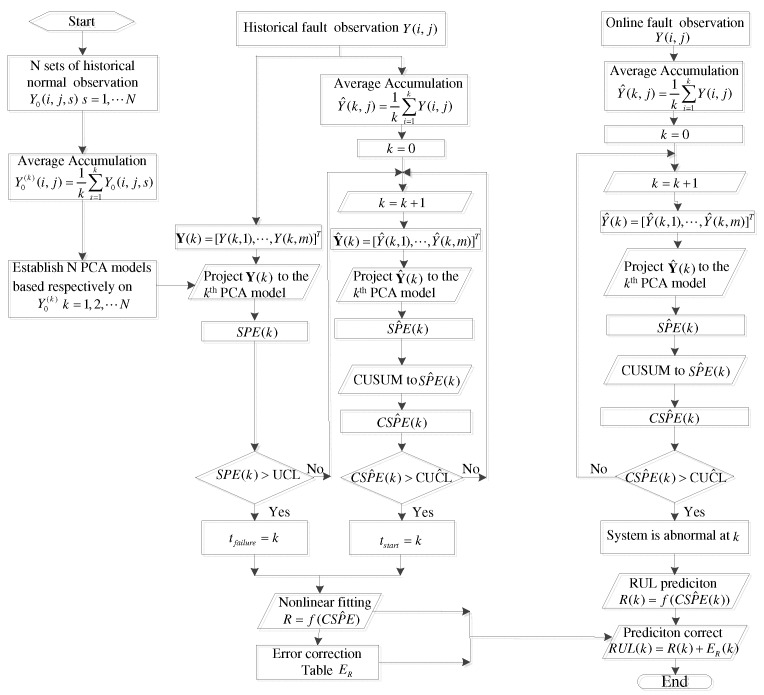
Flowchart of CUSUM-AA-PCA based fault prognosis.

The online error correction term $E_{j,R}\left(C{\hat{W}}_{j}\left(k\right)\right)$ can be determined in a similar form to Equations (40) and (41). The whole fault prognosis process can be scheduled in a flow chart as in Figure 2.

## 5. Simulation

In this part, we illustrate the numeric simulation analysis of AA based time variant model for early diagnosis and prognosis.

The parameters used are m=21, n=512 and l=10. The historical normal observation is generated by the following equation:

(49) $$\begin{array}{c} Y_{0}=\sum_{j=1}^{12}D_{j}{\bar{W}}_{0j}, \end{array}$$

where $D_{j}\;\;\left(j=1,2,\cdots ,12\right)$, defined by Equation (4), are the 12 variation patterns to generate observation data, and ${\bar{W}}_{0j}$ is the *n* samples of ${\bar{w}}_{0j}$ with the distribution $N(0,\;\sigma_{j}^{2})$.

In software, MATLAB, the function "randn" is used to generate ${\bar{W}}_{j}$:

(50) $$\begin{array}{ccc} {\bar{W}}_{01} & = & randn(n,1), \end{array}$$

(51) $$\begin{array}{ccc} {\bar{W}}_{02} & = & 0.5{\bar{W}}_{01}+0.8randn\left(n,1\right), \end{array}$$

(52) $$\begin{array}{ccc} {\bar{W}}_{03} & = & 0.5{\bar{W}}_{01}+0.5{\bar{W}}_{02}, \end{array}$$

(53) $$\begin{array}{ccc} {\bar{W}}_{04} & = & 0.5{\bar{W}}_{02}+0.1randn\left(n,1\right), \end{array}$$

(54) $$\begin{array}{ccc} {\bar{W}}_{05} & = & 0.5{\bar{W}}_{03}+0.2{\bar{W}}_{04}, \end{array}$$

(55) $$\begin{array}{ccc} {\bar{W}}_{06} & = & 0.2{\bar{W}}_{04}+0.3{\bar{W}}_{05}, \end{array}$$

(56) $$\begin{array}{ccc} {\bar{W}}_{07} & = & 0.2randn(n,1), \end{array}$$

(57) $$\begin{array}{ccc} {\bar{W}}_{08} & = & 0.2{\bar{W}}_{07}+0.2randn\left(n,1\right), \end{array}$$

(58) $$\begin{array}{ccc} {\bar{W}}_{09} & = & 0.1{\bar{W}}_{08}+0.2randn\left(n,1\right), \end{array}$$

(59) $$\begin{array}{ccc} {\bar{W}}_{010} & = & 0.2randn(n,1), \end{array}$$

(60) $$\begin{array}{ccc} {\bar{W}}_{011} & = & 0.1{\bar{W}}_{08}+0.2{\bar{W}}_{07}, \end{array}$$

(61) $$\begin{array}{ccc} {\bar{W}}_{012} & = & 0.1{\bar{W}}_{09}+0.1{\bar{W}}_{06}. \end{array}$$

The online normal data are also generated by Equations (50)–(61)

(62) $$\begin{array}{c} Y=\sum_{j=1}^{12}D_{j}{\bar{W}}_{j}. \end{array}$$

The contributions of the fault pattern corresponding to $D_{3}$, $D_{5}$, and $D_{10}$ increase to a large value by

(63) $$\begin{array}{ccc} {\bar{W}}_{3}\left(k\right) & = & {\bar{W}}_{3}\left(k\right)+f_{3}\left(k\right)\;\;k=386,\cdots ,n, \end{array}$$

(64) $$\begin{array}{ccc} {\bar{W}}_{5}\left(k\right) & = & {\bar{W}}_{5}\left(k\right)+f_{5}\left(k\right)\;\;k=33,\cdots ,n, \end{array}$$

(65) $$\begin{array}{ccc} {\bar{W}}_{1}0\left(k\right) & = & {\bar{W}}_{1}0\left(k\right)+f_{1}0\left(k\right)\;\;k=257,\cdots ,n, \end{array}$$

where the fault signal is samples of negative exponential function or linear polynomial function with small slope characterized by

(66) $$\begin{array}{ccc} f_{3}\left(t\right) & = & A_{3}\left(1-e^{-t/\tau_{3}}\right),t\in \left[0,\;\;9\right], \end{array}$$

(67) $$\begin{array}{ccc} f_{5}\left(t\right) & = & A_{5}\left(1-e^{-t/\tau_{5}}\right),t\in \left[5,\;\;16\right], \end{array}$$

(68) $$\begin{array}{ccc} f_{10}\left(t\right) & = & A_{10}t^{\tau_{10}},t\in \left[0.0006,\;\;3\right], \end{array}$$

with the parameter value $A_{3}=15,\;\tau_{3}=30$, $A_{5}=7,\;\tau_{5}=50$, and $A_{10}=0.1,\;\tau_{10}=2.5$.

It can be easily seen from Equations (63)–(68) that every fault occurs at different times and each has a different degradation rate.

### 5.1. AA-Based Time Variant Early Detection of Slowly Varying Small Faults

#### 5.1.1. AA-PCA Early Detection

**Figure 3:**
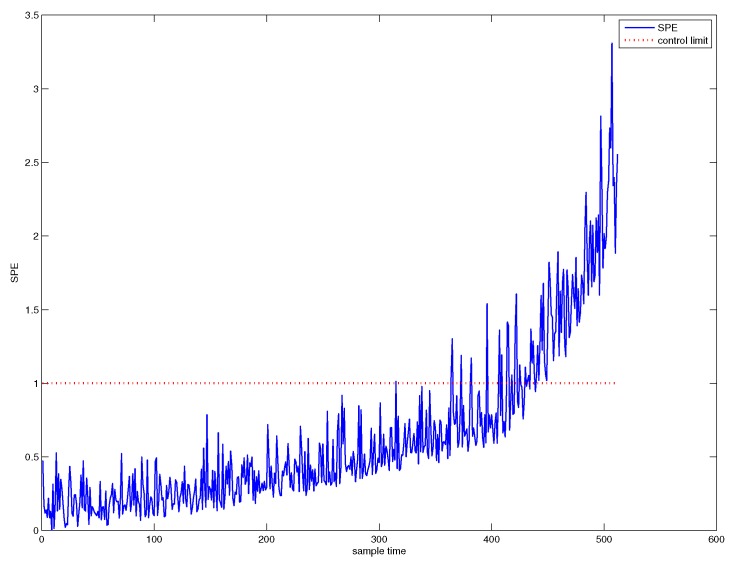
SPE chart for PCA.

First, traditional PCA is used for system monitoring. The SPE chart of PCA is shown in Figure 3, from which from which it can be seen that the system is abnormal from the 425th sample point. The confidence level we have chosen is 1−α=0.997, that is to say, the system is abnormal in the sense of very high confidence level. Hence, we call this fault detection point as the system failure point.

On the other hand, we can see from Figure 3 that a slowly varying fault has occurred in the system from a moment much earlier than the failure point. Before the failure point, the fault is not significant enough to be detected.

CUSUM-PCA is one of the most commonly used early detection methods since it can cumulate fault size beyond a tighter threshold.

**Figure 4:**
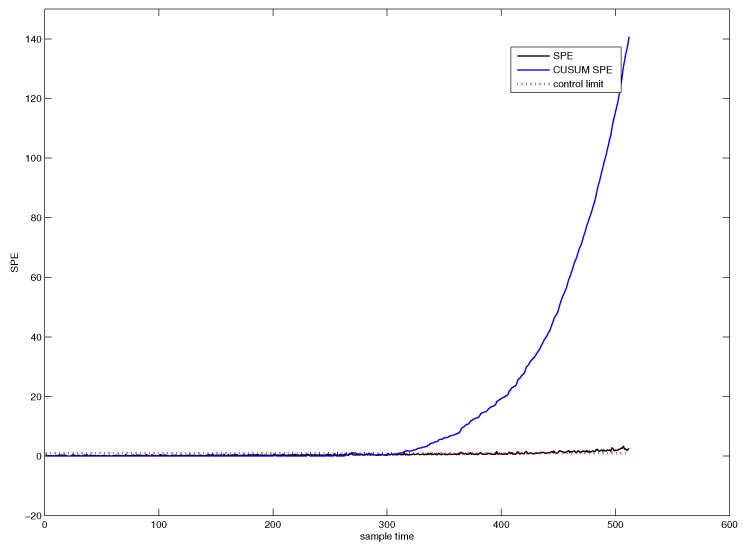
SPE chart for CUSUM-PCA.

Figure 4 shows the SPE chart for CUSUM-PCA. The blue line denotes the SPE value of CUSUM-PCA, and the black line represents the SPE value of PCA. It can be seen from Figure 4 that the system has followed an abnormal trend from the 311th sample point.

**Figure 5:**
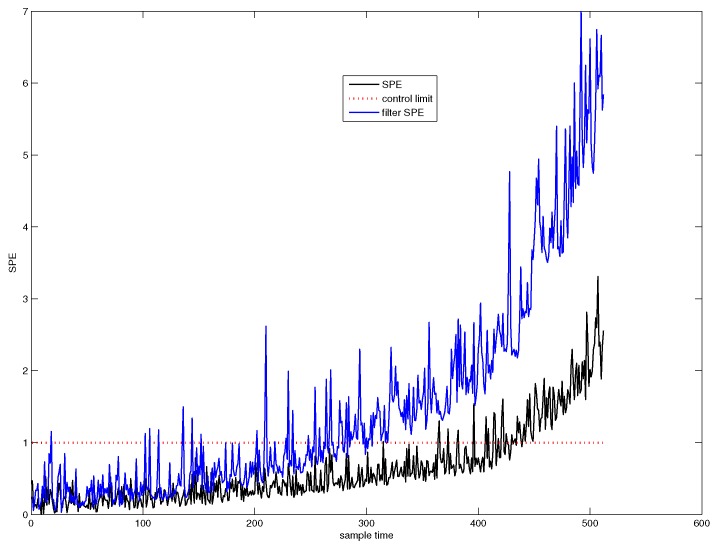
SPE chart for WF-PCA.

The SPE chart of wavelet filter (WF) based PCA is depicted in Figure 5. The blue line denotes the SPE value of wavelet filter based PCA (WF-PCA), and the black line denotes the SPE value of PCA. It can be seen from Figure 5 that the system has followed an abnormal trend from the 281th sample point.

**Figure 6:**
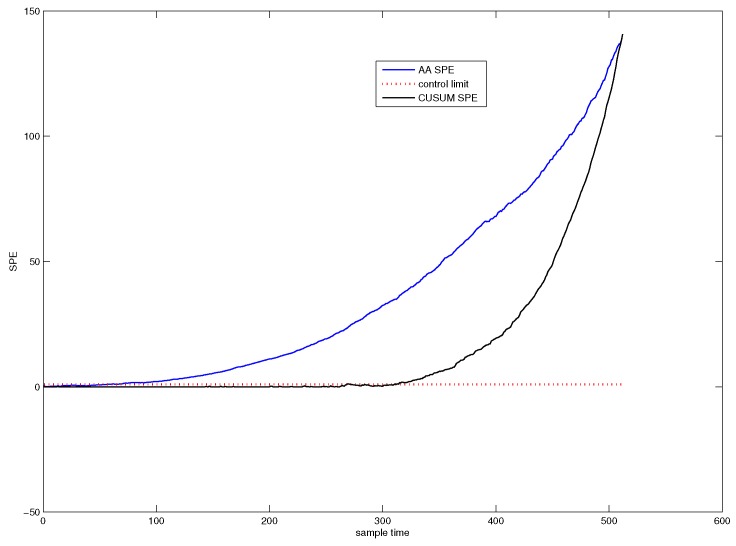
SPE chart for AA-PCA.

As we have analyzed in Section 1, either filter based method to decrease noise energy or the CUSUM based method to cumulate the fault size can not detect the fault trend well as early as possible. Figure 6 illustrates the early fault detection result of time variant AA-PCA. It tells us that the fault trend can be detected at the 57th sample point.

It can also be seen from Figure 6 that the fault trend has occurred before the detection point $t_{0}=57$. Since the noise energy has been reduced to a rather small level, so the CUSUM technique can be used for the SPE chart of time variant AA-PCA.

**Figure 7:**
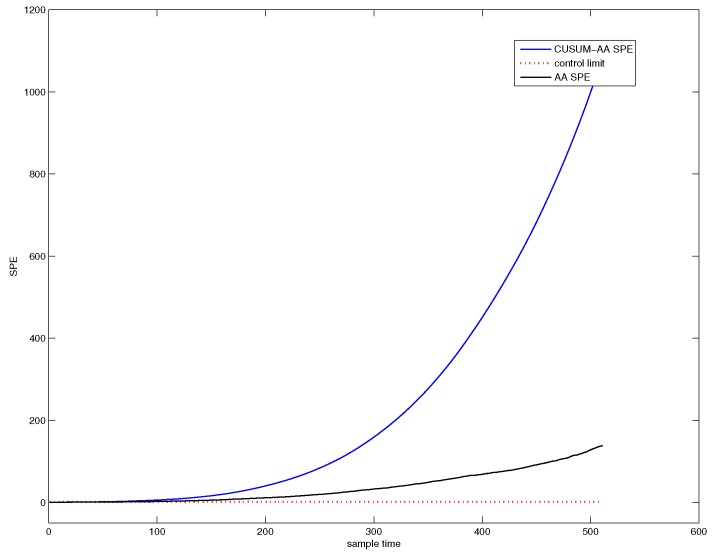
SPE chart for CUSUM-AA-PCA.

Figure 7 shows the detection efficiency of the CUSUM-AA-PCA method. The blue line denotes the SPE value of CUSUM-AA-PCA, and the black line denotes the SPE value of AA-PCA. Figure 7 indicates that the system has started an abnormal trend from the 44st sample point.

The miss detection rate, false detection rate, and the fault trend detection point corresponding to Figure 3, Figure 4, Figure 5, Figure 6 and Figure 7 are listed in Table 1. From the table, it can be concluded that CUSUM-AA based time variant early fault detection for a slowly varying fault is a more efficient method since it can effectively decrease the noise energy as well as can accumulate the fault size.

#### 5.1.2. AA-DCA Based Early Diagnosis

**Figure 8:**
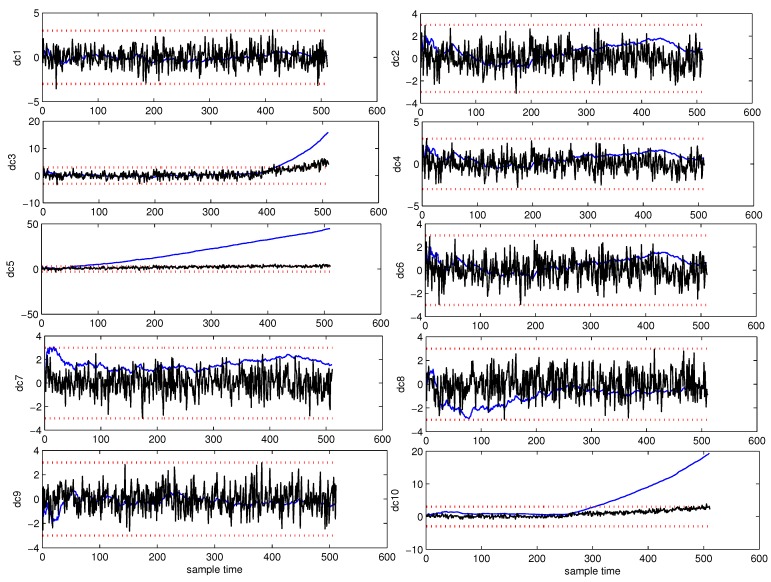
Shewhart chart of AA-DCA.

Affected by the pattern compounding effect, the PCA based fault detection method and its variations can only alert users that the system is abnormal. It can not find the root cause of the fault component. DCA is introduced to overcome this problem. Figure 8 shows the Shewhart chart of AA-DCA diagnosis and DCA diagnosis. The black line represents the Shewhart of DCA diagnosis, the blue line denotes the Shewhart of AA-DCA diagnosis, and the red dotted line are the upper control limit and the lower control limit, respectively.

From Figure 8, it can be seen that three faults corresponding to $D_{3}$, $D_{5}$, and $D_{10}$ have occurred in the system. The detection point of each fault is listed in Table 2.

**Figure 9:**
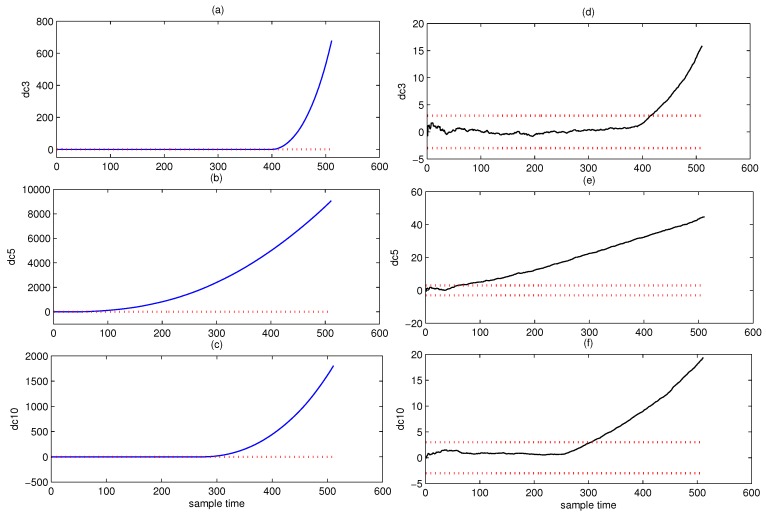
Shewhart to $D_{3}$, $D_{5}$ or $D_{10}$ for CUSUM-AA-DCA. The subfigures (**a**–**c**) are the Shewhart charts to $D_{3}$, $D_{5}$, or $D_{10}$ of CUSUM-AA-DCA. The subfigures (**d**–**f**) are the Shewhart charts to $D_{3}$, $D_{5}$, or $D_{10}$ of AA-DCA. The dotted red line is the control limit.

Figure 9 shows the Shewhart chart of CUSUM-AA-DCA diagnosis. The subfigures (a–c) are the Shewhart charts to $D_{3}$, $D_{5}$, or $D_{10}$ of CUSUM-AA-DCA. The subfigures (d–f) are the Shewhart charts to $D_{3}$, $D_{5}$, or $D_{10}$ of AA-DCA. The dotted red line is the control limit.

From Figure 9, it can be seen that CUMSUM-AA-DCA can diagnose faults at earlier times than that of AA-DCA. The miss detection rate, false detection rate, and the fault trend detection point of different method are listed in Table 2. Comparing the 2nd row, the 6th row and 10th row of Table 2 with the 2nd row of Table 1, it can be concluded that the detection point of the SPE chart for PCA is not equal to any of the detection point of $D_{3}$, $D_{5}$, or $D_{10}$, which implies that PCA can not effectively detect the root cause of the faults.

Comparing Table 2 with Table 1, it can be concluded that CUSUM-AA based time variant early diagnosis for slowly varying faults is more efficient.

### 5.2. Fault Prognosis

In this part, we first use the early fault detection result based respectively on CUSUM-AA-PCA and CUSUM-AA-DCA of the historical fault observation to establish the damage precursor and the RUL prediction model. Then, online RUL can be predicted.

**Remark** **8.** *Although an AA based time variant model is the foundation of this paper, in order to save space in this paper, only CUSUM-AA based RUL prediction is illustrated in this part since CUSUM-AA can detect slowly varying faults at earlier times ahead of failure.*

#### 5.2.1. RUL Prediction Model Based on Historical Faulty Observation

**Figure 10:**
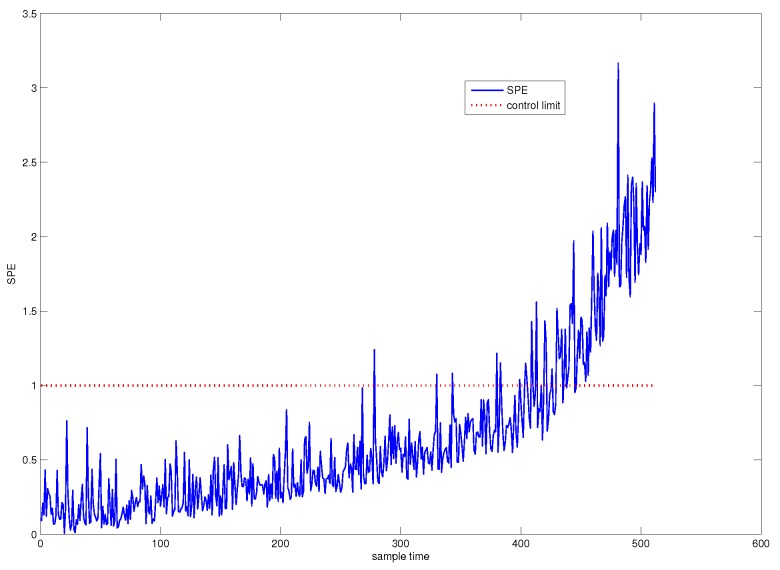
SPE chart for PCA for historical fault observation.

For the purpose of establishing an RUL prediction model, a damage precursor characterizing the fault size evolving process should first be determined. In this subsection, the statistics value characterizing fault size is SPE of CUSUM-AA-PCA. Implement PCA into the historical fault observation, and display the SPE chart in Figure 10. Using this control chart to determine the failure point, $t_{failure}=421$.

**Figure 11:**
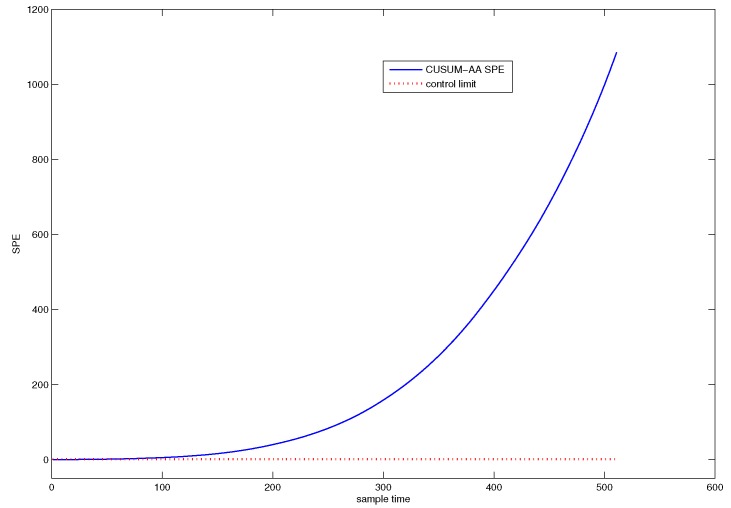
SPE chart for CUMSUM-AA-PCA to historical fault observation.

By implementing CUSUM-AA-PCA into the historical fault observation, the corresponding SPE chart can be shown in Figure 11. We can use this chart to determine the starting point $t_{start}=44$.

The obtained SPE value for CUSUM-AA-PCA from $t_{start}$ to $t_{failure}$ can be defined as the accumulated damage precursor.

**Figure 12:**
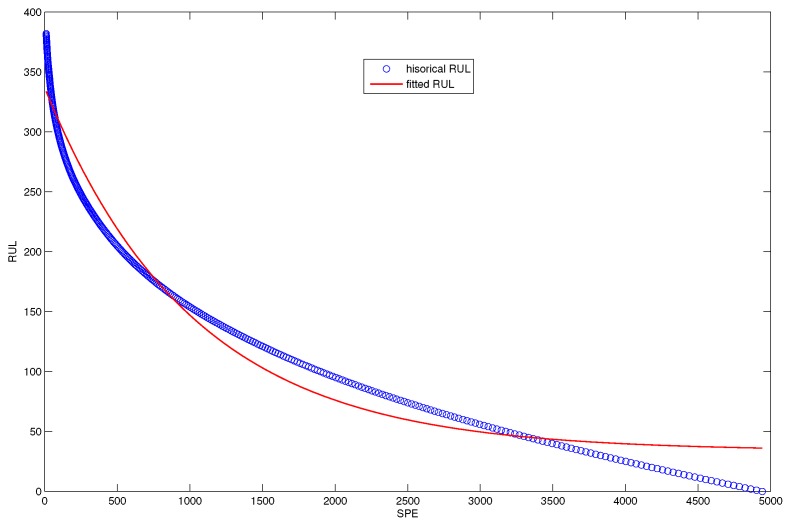
The fitted RUL prediction model based on the SPE damage precursor.

Figure 12 shows the nonlinear fitting curve of the accumulated damage precursor. The blue scatter chart denotes the real historical RUL and the red line denotes fitted RUL.

**Figure 13:**
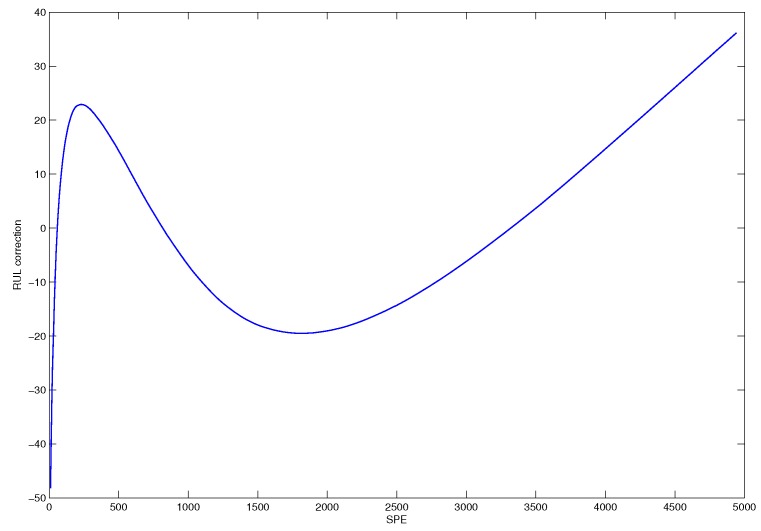
The error correction curve of SPE based prediction model.

The error correction curve illustrated in Figure 13 can be used to establish a error correction table for the prediction model.

**Figure 14:**
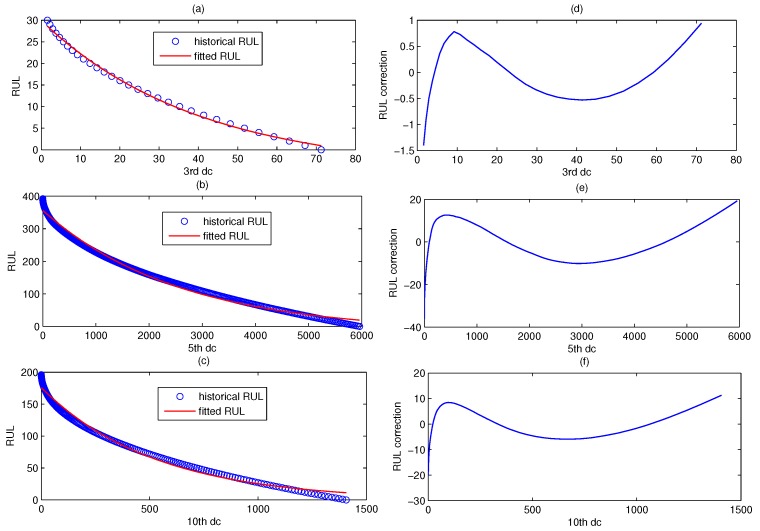
The fitted RUL prediction model based on DC corresponding to $D_{3}$, $D_{5}$ and $D_{10}$. The left part (**a**–**c**) is the fitted RUL prediction model for the three fault patterns corresponding to $D_{3}$, $D_{5}$, and $D_{10}$, and the right part (**d**–**f**) is their model error correction curve.

Figure 14 shows the fitted RUL prediction model of CUSUM-AA-DCA. The blue scatter chart denotes the real historical RUL, and the red line denotes fitted RUL. The left part is the fitted RUL prediction model for the three fault patterns corresponding to $D_{3}$, $D_{5}$, and $D_{10}$, and the right part is their model error correction curve.

### 5.3. Online RUL Prediction

Once online $CS\hat{P}E\left(k\right)$ of CUSUM-AA-PCA is obtained, use Equation (38) to predict the online RUL(k).

**Figure 15:**
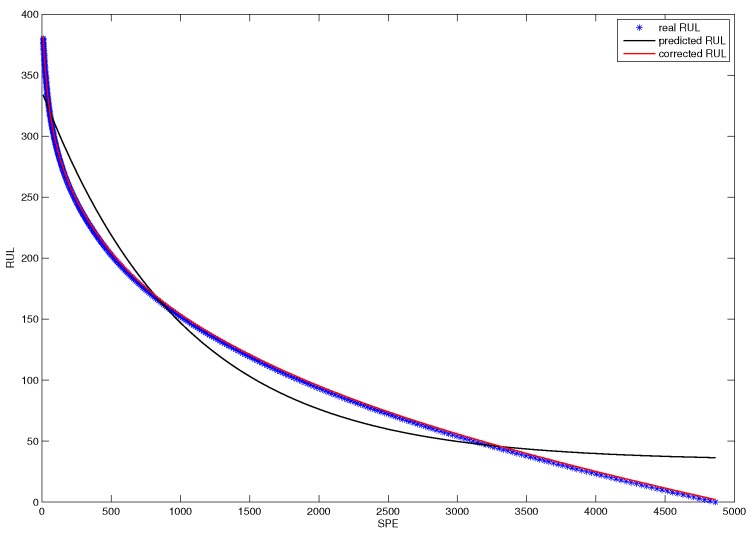
Online RUL prediction based on SPE.

Figure 15 shows the online RUL prediction result of the CUSUM-AA-PCA fault prognosis method. The scatter chart denotes the real RUL, the black line represents the rough predicted RUL, and the blue line is the corrected RUL.

**Figure 16:**
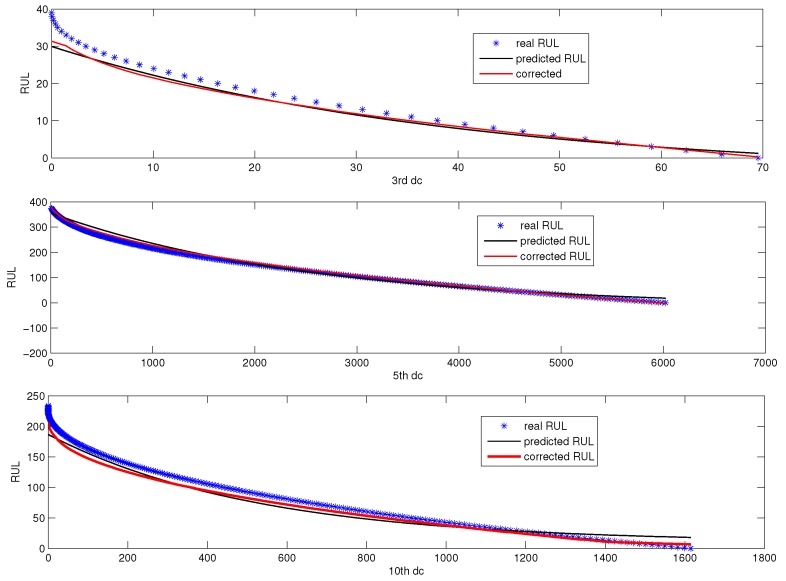
Online RUL prediction based on DC corresponding to $D_{3}$, $D_{5}$ and $D_{10}$.

Figure 16 is the online RUL prediction based on the three designated components corresponding to $D_{3}$, $D_{5}$, and $D_{10}$. The scatter chart denotes the real RUL, the black line is the rough predicted RUL, and the blue line is the corrected RUL.

**Figure 17:**
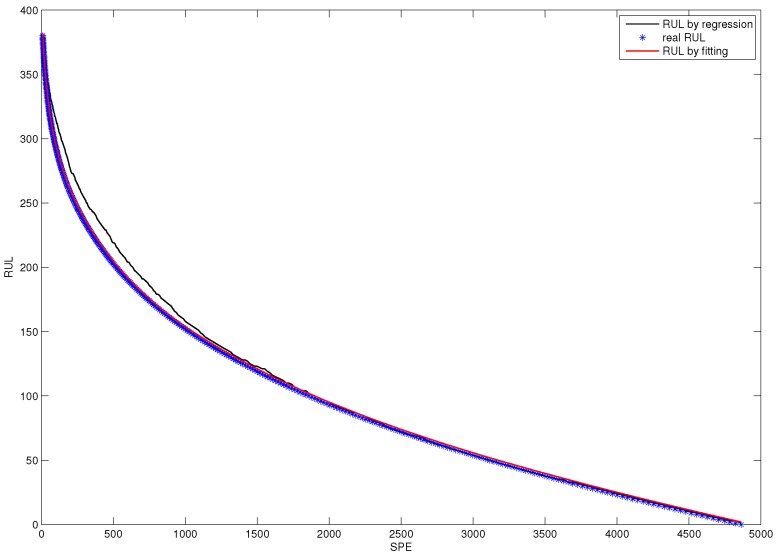
RUL prediction using AR to SPE.

**Figure 18:**
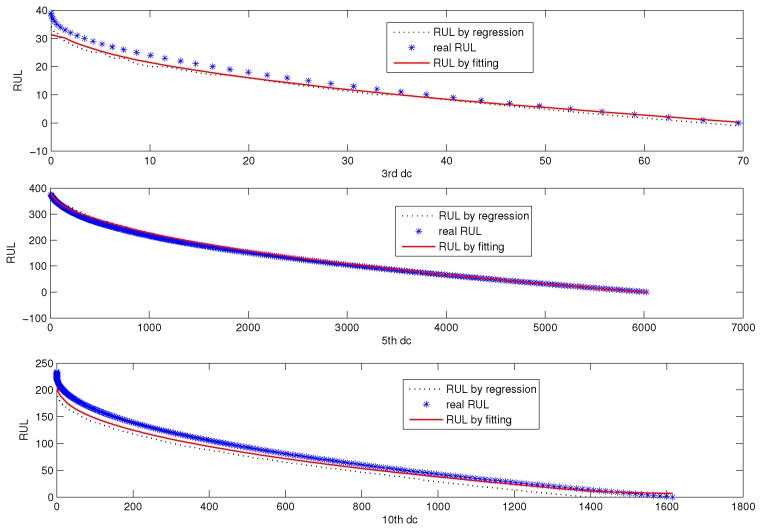
RUL prediction using AR to DC corresponding to $D_{3}$, $D_{5}$ and $D_{10}$.

Figure 15 and Figure 16 tell us that the proposed RUL prediction model with error correction is effective for fault prognosis in the sense that RUL prediction error is small. Figure 17 shows the online RUL prediction of AutoRegressive (AR) following recursive RUL estimation. Figure 18 is the RUL prediction using AR to DC corresponding to $D_{3}$, $D_{5}$, and $D_{10}$. It can be seen from Figure 18 that the fitted RUL prediction model with error correction is more efficient than that of the AR model.

The mean of absolute RUL prediction error is listed in Table 3. From Table 3, it can be concluded that the RUL prediction model with error correction is a better choice in the case when only one set of fault observations is available.

**Remark** **9.** *If the variance of the statistics characterizing fault size is larger, the efficiency of the fitted prediction model with error correction will be more superior to that of the AR prediction model. The reason is that the AR prediction model is a local linear model, and error propagation is significant during the recursive regression process.*

## 6. Conclusions

Condition-based maintenance is one of the necessary means to ensure safety and economic production. The purpose of fault prognosis is to judge whether a fault will occur and how long the system will break down in the future, which is the foundation of condition-based maintenance. Early detection of slowly varying fault is an essential step for developing an accurate RUL prediction model.

In this paper, we first propose a novel AA based time variant PCA model for early detection of slowly varying small faults, which can accumulate fault size as well as decrease the noise energy. DCA is introduced to develop an AA based time variant DCA to diagnose the root cause of the fault, which is helpful for the operator to make decisions about system maintenance. Then, the AA based method is combined with the CUSUM based method to detect fault trends at earlier times. Finally, the RUL prediction model with error correction is established by nonlinear fitting. Once the online fault size defined by a detection statistics value is obtained by an early diagnosis algorithm, the online RUL can be predicted by this fitting curve and the error correction table.

In the case of many sets of fault observations being available, deep learning based on early detection and prognosis of slowly varying faults will be our future focus.

## Figures and Tables

**Table 1:** Detection result of SPE chart for PCA.

Method	Detection Point	FDR	MDR
PCA	421	0%	81.0412%
WF-PCA	281	3.125%	51.875%
CUMSUM-PCA	311	0%	58.125%
AA-PCA	57	0%	5.2083%
CUMSUM-AA-PCA	44	0%	2.5%

**Table 2:** Detection results of the Shewhart chart for DCA.

Statistics	Method	Detection Point	FDR	MDR
DC3	DCA	480	4.6875%	75%
	CUMSUM-DCA	419	0%	27.3438%
	AA-DCA	416	0%	25%
	CUMSUM-AA-DCA	403	0%	14.8438%
DC5	DCA	470	0.7813%	91.25
	CUMSUM-DCA	201	0%	35.2083%
	AA-DCA	60	0%	5.8333%
	CUMSUM-AA-DCA	52	0%	4.1667%
DC10	DCA	512	0%	99.6094
	CUMSUM-DCA	404	0%	57.8125%
	AA-DCA	305	0%	19.1406%
	CUMSUM-AA-DCA	278	0%	8.5938%

**Table 3:** Mean of absolute RUL prediction error.

Statistics	Exponential Fittting	Auto-Regressive
SPE	2.0000	9.8084
DC3	2.1846	2.5500
DC5	6.5660	6.6320
DC10	11.5484	19.6766
